# Optical Monitoring of Breathing Patterns and Tissue Oxygenation: A Potential Application in COVID-19 Screening and Monitoring

**DOI:** 10.3390/s22197274

**Published:** 2022-09-26

**Authors:** Aaron James Mah, Thien Nguyen, Leili Ghazi Zadeh, Atrina Shadgan, Kosar Khaksari, Mehdi Nourizadeh, Ali Zaidi, Soongho Park, Amir H. Gandjbakhche, Babak Shadgan

**Affiliations:** 1 Implantable Biosensing Laboratory, ICORD, Vancouver, BC V5Z 1M9, Canada; 2 Department of Pathology & Laboratory Medicine, University of British Columbia, Vancouver, BC V6T 1Z7, Canada; 3 Eunice Kennedy Shriver National Institute of Child Health and Human Development, National Institute of Health, Rockville, MD 20847, USA; 4 Department of Orthopedics, University of British Columbia, Vancouver, BC V6T 1Z7, Canada; 5 Department of Biomedical Engineering, University of British Columbia, Vancouver, BC V6T 1Z7, Canada

**Keywords:** COVID-19 screening, COVID-19 monitoring, pneumonia, respiratory disease, NIRS, optical monitoring, wearable biosensor, respiratory monitoring, breathing patterns, tissue oxygenation

## Abstract

The worldwide outbreak of the novel Coronavirus (COVID-19) has highlighted the need for a screening and monitoring system for infectious respiratory diseases in the acute and chronic phase. The purpose of this study was to examine the feasibility of using a wearable near-infrared spectroscopy (NIRS) sensor to collect respiratory signals and distinguish between normal and simulated pathological breathing. Twenty-one healthy adults participated in an experiment that examined five separate breathing conditions. Respiratory signals were collected with a continuous-wave NIRS sensor (PortaLite, Artinis Medical Systems) affixed over the sternal manubrium. Following a three-minute baseline, participants began five minutes of imposed difficult breathing using a respiratory trainer. After a five minute recovery period, participants began five minutes of imposed rapid and shallow breathing. The study concluded with five additional minutes of regular breathing. NIRS signals were analyzed using a machine learning model to distinguish between normal and simulated pathological breathing. Three features: breathing interval, breathing depth, and O_2_Hb signal amplitude were extracted from the NIRS data and, when used together, resulted in a weighted average accuracy of 0.87. This study demonstrated that a wearable NIRS sensor can monitor respiratory patterns continuously and non-invasively and we identified three respiratory features that can distinguish between normal and simulated pathological breathing.

## 1. Introduction

The recent outbreak of coronavirus disease 19 (COVID-19), caused by severe acute respiratory syndrome coronavirus 2 (SARS-Cov-2), has placed a spotlight on techniques for early screening, detection, and monitoring of respiratory disease in acute and chronic phases. At the beginning of the COVID-19 pandemic, a lack of an accurate and rapid screening technique was a significant issue contributing to the spread of the virus [1]. Currently, the most commonly used method for determining COVID-19 infection is using molecular assays such as reverse transcriptase–polymerase chain reaction (RT-PCR). However, RT-PCR tests typically take hours to determine results, and in specific incidences of mass infection, the turnaround time can take three days or longer [2]. Furthermore, there are questions regarding the sensitivity and specificity of the test, with false-negative test results being a major concern [3]. An alternative to RT-PCR testing is antigen–detection rapid diagnostic tests (Ag-RDTs) to diagnose an active infection. However, these tests tend to be less accurate than molecular assays and report a high number of false-negative tests [4]. More importantly, these tests are not monitoring techniques, and they only provide information about the presence of the virus, but not the infected patients’ health condition. Therefore, a rapid, non-invasive, and inexpensive method for the periodic regular screening and early diagnosis of acute pneumonia, such as in COVID-19 patients, is crucially needed to prevent the spread of infection and improve patient health, particularly in high-risk individuals.

When viral pneumonia develops in COVID-19 patients, it initially causes a form of oxygen deprivation called ‘silent hypoxia’. In this situation, tissue oxygen levels start to drop silently without other overt symptoms. Patients then gradually begin to compensate for the hypoxia by breathing faster and shallower [5]. This causes tissue hypoxia and hyperventilation, and the arterial oxygen saturation (SpO_2_) level begins to drop, causing hypoxemia. Conventionally, hypoxemia is identified by standard pulse oximeters (SpO_2_ < 90%) [6]. In COVID-19, hypoxemia precipitates more inflammation and damage to the lung tissue by forcing the patient to breathe harder and deeper, which leads to a second and deadlier phase of lung injury [7]. Measuring SpO_2_ levels by pulse oximetry may not be the most reliable method for early detection of COVID-19 [8,9,10,11] due to its technical and physiological limitations, including low accuracy. This can be particularly true in complicated cases such as severely ill and hypotensive patients and those who take certain medications [6,12]. 

In addition to hypoxemia, individuals with acute pneumonia experience abnormal breathing patterns soon after infection starts to develop. The breathing pattern encompasses respiratory rate, rhythm, and effort. The normal resting respiratory rate is 12 to 20 breaths per minute, with an estimated 1:3 ratio of inspiration to expiration duration [13]. Patients with acute pneumonia initially experience breathing difficulty, causing cough with rapid and shallow breathing (tachypnoea) [14]. As pneumonia progresses, the lungs become inflamed, and the alveolar spaces fill with inflammatory exudate and sputum. This leads to loaded breathing, causing the patients to increase their ventilatory efforts [15]. Hyperventilation is a critical sign of highly damaged lung tissue when patients require respiratory support. If not controlled, patients enter a dangerous and life-threatening condition when their respiratory centers become depressed, leading to low-frequency and shallow breathing (bradypnea) [16]. These complications can be prevented if the respiratory disease is detected and treated early. Screening and diagnosis of acute pneumonia based on clinical features or measuring SpO_2_ can be unreliable, misleading, and late. An accurate and reliable method for continuous monitoring of pulmonary function in patients with acute pneumonia could provide several benefits. These include triage of infected patients based on disease severity, monitoring disease progression and responses to treatment, and developing timelines for discharge from the hospital. An accurate and noninvasive technique that identifies changes in the respiratory pattern can be used for screening, early diagnosis, and monitoring of patients with acute pneumonia, including COVID-19. 

Due to the novel nature of COVID-19, much remains unknown regarding the long-term effects of the disease. There is evidence that specific individuals infected with COVID-19 may develop long-term respiratory complications [17]. Therefore, longitudinal monitoring of pulmonary health and function following an infection can provide important information regarding disease remission or progression in the chronic phase. A method for continuously monitoring respiratory function could provide advantages over clinical tests such as spirometry, chest radiography, and scans that require trained personnel, as well as visits to a specialized facility, which can impose a significant financial burden on patients and healthcare resources [18,19].

Near-infrared spectroscopy (NIRS) is an optical technology that utilizes light in the near-infrared spectrum (650–1000 nm) to provide continuous measurements of tissue hemodynamics and oxygenation [20]. NIRS measures the change in concentration of tissue oxygenated (O_2_Hb) and deoxygenated hemoglobin (HHb) chromophores, which can provide real-time information about tissue oxygenation status [20]. The sum of O_2_Hb and HHb, called total hemoglobin (THb), is a blood volume parameter within the tissue. Using multiple light sources and photodetectors configured in a spatially resolved configuration, NIRS sensors can also provide an absolute measure of tissue oxygen saturation level (TSI) [21,22]. TSI provides a measure of the ratio of O_2_Hb to THb and can be helpful in comparing different conditions [21,22]. 

NIRS has been well-documented as a useful monitoring tool in several clinical applications in trauma, neurology, urology, musculoskeletal medicine, and sports medicine [20,23,24]. Several researchers have used this technique to evaluate respiratory muscle metabolism in health and disease [25,26,27]. However, there is limited research on the effectiveness of NIRS for monitoring respiratory function in patients with pneumonia, particularly COVID-19 disease, as a screening, diagnostic, and monitoring tool. The non-invasive nature and ability to provide real-time, continuous monitoring makes NIRS an attractive technology for monitoring respiratory infectious diseases such as COVID-19. A common feature in individuals with acute respiratory disease is the development of hypoxia and hypoxemia, disrupting the body’s oxygenation [28]. Several respiratory and cardiac parameters, such as breathing rate, breathing depth, and heart rate, can be extracted by analyzing NIRS signals taken from the chest transcutaneously. In combination with additional physiological data such as body temperature and arterial oxygen saturation (SpO_2_), NIRS has the potential to detect and monitor abnormal respiration patterns or reduced tissue and systemic oxygenation seen in individuals with respiratory disease [28]. Therefore, we hypothesize that developing a multi-modal biosensor containing a NIRS sensor can be highly beneficial to screening, diagnosing, and monitoring infectious respiratory diseases in the acute and chronic phases. 

### Objective

This study aimed to examine the feasibility and efficacy of using a wearable NIRS sensor to collect respiratory signals and distinguish between normal and simulated pathological breathing. The outcomes of this study will contribute to developing a multi-modal biosensor for the early detection of individuals with acute pneumonia, such as in COVID-19 patients. 

## 2. Methods

In this study, we examined the ability of a wearable NIRS sensor placed on the chest of a participant to collect respiratory signals in response to a simulated pathological breathing protocol. We then applied a machine learning model to distinguish between normal and simulated pathological breathing.

### 2.1. Instrumentation

**Figure 1:**
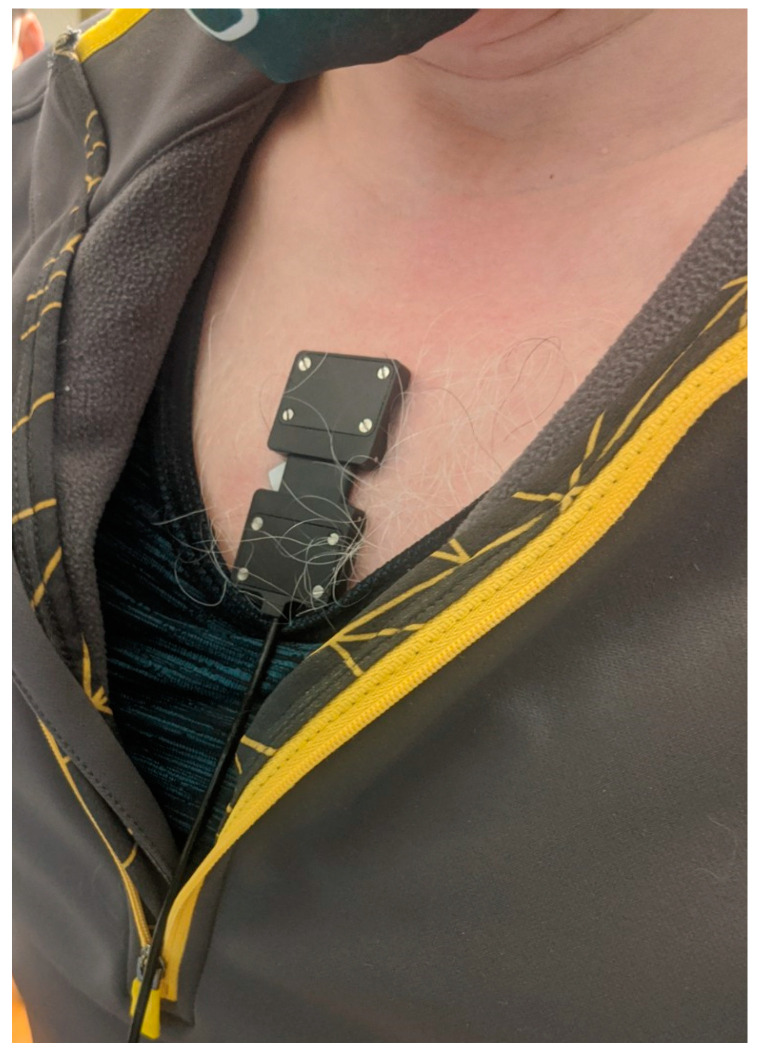
NIRS sensor affixed over the sternal manubrium.

This study used a compact, continuous-wave, wearable NIRS sensor (PortaLite, Artinis Medical Systems, BV, Netherlands). The NIRS sensor contains three light sources, each with two light-emitting diodes (LED)s emitting light at wavelengths of 760 nm and 850 nm. The three sources are located at distances of 30 mm, 35 mm, and 40 mm from the photodetector, enabling the acquisition of spatially resolved NIRS data. Data is collected at a sampling rate of 10 Hz and transmitted via Bluetooth^®^ to a laptop computer containing software for data acquisition, visualization, analysis, and storage. The NIRS software allows visualization of O_2_Hb, HHb, THb and TSI in real time. The NIRS sensor was affixed to the participant’s sternum using double-sided tape (Figure 1).

**Figure 2:**
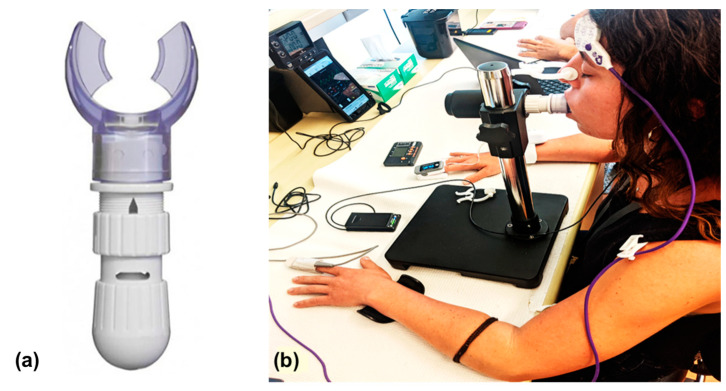
Respiratory trainer (**a**) and the apparatus (**b**) used during the loaded breathing stage.

To simulate loaded inspiration, which occurs in acute pneumonia, we used a respiratory trainer (Ultrabreathe Respiratory Trainer, Tangent Healthcare, Market Drayton, United Kingdom). The respiratory trainer increases the resistance during inspiration, causing the respiratory muscles to exert more effort to breathe. The respiratory trainer was set to the highest resistance possible for participants to breathe comfortably for five minutes. The device was attached to a lever placed at the height of the participant’s mouth, allowing them to access the respiratory trainer comfortably and with limited movement (Figure 2). A pulse oximeter (Caretaker Medical, Charlottesville, VA, USA) was attached to the right index finger of participants to monitor SpO_2_, and a thermometer sensor (3M Bair Hugger Temperature Monitoring System, 37000, 3M Medical, St. Paul, MN, USA) was attached to the forehead of participants to monitor body temperature in real-time [29]. These two sensors were used as a safety precaution to monitor the participants during the experiment.

### 2.2. Participants

A group of 21 healthy young adults (12 male, 9 female) with a mean age of 29 years was recruited for the study. Participants were included in the study if they were healthy adults between the ages of 18–65 and excluded if they had a history of cardiopulmonary conditions or anxiety. The Clinical Research Ethics Board at the University of British Columbia approved the study’s protocol, and all participants provided informed consent at the beginning of a data collection session.

### 2.3. Experimental Protocol

**Figure 3:**
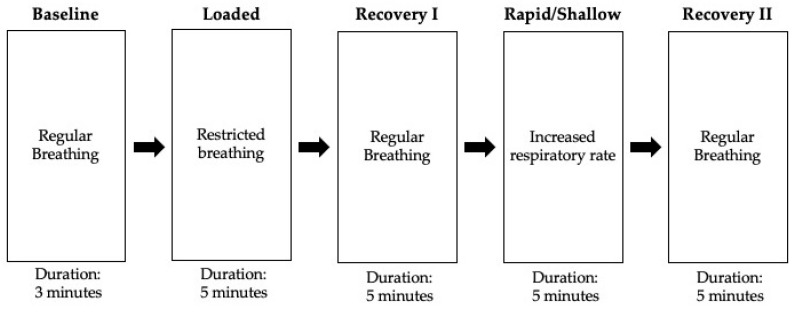
Flowchart describing the experimental protocol.

We designed the protocol to collect respiratory signals using a compact, wearable NIRS sensor while participants underwent five separate breathing conditions (Figure 3). Prior to the start of the experiment, participants sat in a resting position for a 15 min period to limit the effect of external factors such as elevated heart rate and temperature on NIRS data. The NIRS sensor was placed over the sternum (sternal manubrium) to record respiratory signals transcutaneously throughout the 23 min experiment. The sensor was then covered with a black cloth to reduce the effect of ambient light on the NIRS signals. The participants sat with their hands at navel height throughout the experiment and the pulse oximeter was placed on the left index finger. The thermometer sensor was placed on the forehead to monitor body temperature (Figure 2). 

The study protocol is illustrated in Figure 3. During an experiment, participants were instructed to remain quiet and restrict movement to limit motion artifacts on the NIRS signals. Participants were monitored closely by two trained research assistants and were instructed to raise their hands and stop the experiment if any symptoms such as dizziness, light-headedness, or headache were experienced. Additionally, the experiment was stopped if the SpO_2_ decreased below 90% or if the body temperature was elevated above 38 °C. However, this did not occur in any subjects during this study.

Participants began the first stage of the experiment sitting in a resting position while breathing normally through the mouth or nose for a 3 min period to establish a baseline. Following the baseline stage, participants began a 5 min period of breathing using a respiratory trainer (loaded breathing), restricting the participants’ ability to inhale. A clip was placed over the nose to restrict nasal breathing. The loaded breathing stage was designed to simulate dyspnea, a shortness of breath commonly seen in individuals with viral pneumonia. Participants conducted five minutes of regular breathing in the first recovery stage following the loaded breathing stage. Participants were then instructed to increase their respiratory rate and take rapid, shallow breaths for a 5 min period during the next stage. A metronome was used to maintain a respiratory rate of 25 breaths per minute. The rapid, shallow breathing stage was designed to simulate tachypnea, an inability to take a deep breath that develops during acute pneumonia. The experiment concluded with five minutes of regular breathing during the second recovery stage. 

### 2.4. Data Pre-Processing

**Figure 4:**
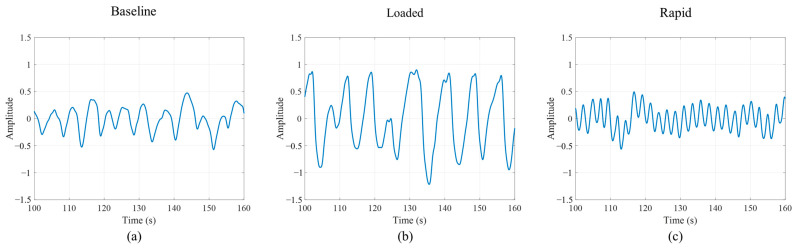
O_2_Hb signal amplitude during three breathing conditions; (**a**) baseline, (**b**) loaded breathing, and (**c**) rapid breathing.

Data pre-processing was performed using customized MATLAB (R2021a, MathWorks) code. Data collected for a 23 min period were divided into five conditions (baseline, loaded breathing, recovery 1, rapid breathing, and recovery 2). Figure 4 displays a segment (60 s) of O_2_Hb signal amplitude change during each breathing condition. At our sampling rate of 10 Hz, respiration and pulse are clearly visible in the NIRS signal (Figure 4). 

### 2.5. Data Analysis

In this study we simulated breathing in viral pneumonia by using a loaded breathing task, and used respiratory function parameters such as breathing interval, breathing depth, and O_2_Hb signal amplitude as features for our classifier. Breathing interval and breathing depth were extracted from O_2_Hb signals using a peak detection procedure with custom parameters detailed below. 

#### 2.5.1. Peak Detection

**Figure 5:**
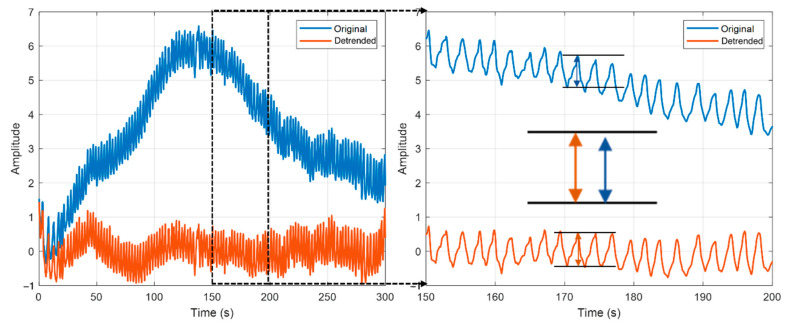
Data detrending: original O_2_Hb signals were detrended to remove large trends.

First, the O_2_Hb signal was detrended by fitting a 6th order polynomial to the original signal and subtracting it to remove large-scale trends in the data not relevant to the analysis (Figure 5). After that, we determined the average distance between any two consecutive peaks. The average distance was then passed as a parameter to a MATLAB’s findpeaks function to detect the peak-maximum. The peak-minimum was similarly determined by inverting O_2_Hb signal calling findpeaks again. Consecutive maxima or minima, which did not have a corresponding minimum/maximum peak in between, were removed from the analysis.

#### 2.5.2. Feature Selection

**Figure 6:**
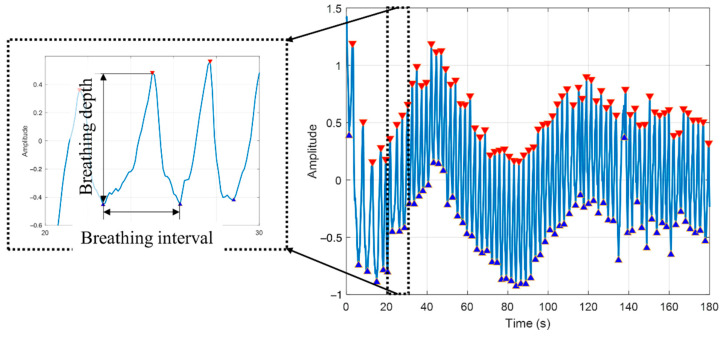
Peak detection and breathing interval and depth calculation.

The breathing interval was calculated as the time difference between two consecutive peak minima (Figure 6). Breathing depth was the amplitude difference between a peak maximum and its previous peak minimum (Figure 6). The mean and standard deviation of the breathing interval were calculated for each participant in each condition. The breathing interval and its corresponding breathing depth that had a value falling out of the 95% confidence interval were excluded from our analysis.

#### 2.5.3. Machine Learning

Breathing interval, breathing depth, and O_2_Hb signal amplitude changes from each participant were divided into 1 min windows for each condition, and then averaged, resulting in 3 datapoints for baseline, 5 datapoints for loaded breathing, and 5 datapoints for rapid breathing. Random forest classification, a well-known supervised machine-learning technique, was used to classify the three conditions [30]. The model included 100 trees with no maximum depth per tree. The complete dataset was randomly partitioned with an 80:20 ratio for model training and evaluation respectively. This was repeated 100 times with different randomly selected training and testing sets. Reported accuracy is the average accuracy of these 100 validation runs.

## 3. Results

**Figure 7:**
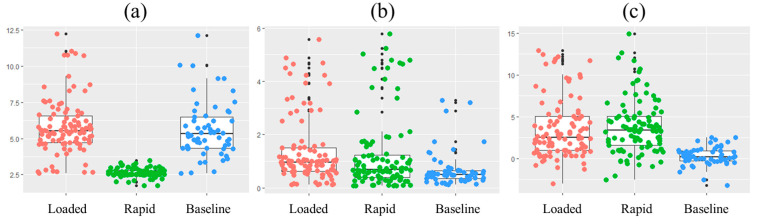
Selected features in each condition: (**a**) breathing interval; (**b**) breathing depth; and (**c**) O_2_Hb signal amplitude.

In general, the breathing interval had the lowest mean and smallest variation during the rapid breathing condition (Figure 7). This is not surprising since all subjects were told to breathe at a certain rate. However, during the baseline and loaded breathing conditions, when a breathing rate was not enforced, the variation was larger due to individual differences. Breathing depth and O_2_Hb signal amplitude were lowest during the baseline and higher in the other two conditions. Classification of the three conditions using three features (breathing interval, breathing depth, and O_2_Hb signal amplitude) resulted in a weighted average accuracy of 0.87 and F1-score of 0.86. 

When only two of three features were used, the weighted average accuracy and F1-score decreased to 0.79 and 0.77 (breathing interval and breathing depth), 0.78 and 0.76 (breathing interval and O_2_Hb signal amplitude), and 0.63 and 0.62 (breathing depth and O_2_Hb signal amplitude), respectively. A summary of the mean accuracy using different respiratory features is listed in Table 1. 

## 4. Discussion

The COVID-19 pandemic has highlighted the need for a novel method to continuously monitor respiratory function in the acute and chronic phases. In people infected by acute viral pneumonia, including COVID-19 patients, alveolar lung cells are attacked, and gradually destroyed, by the viral infection leading to hypoxia, hypoxemia, and lung dysfunction [31,32]. Infected patients can develop chest discomfort, dyspnea, and other progressive respiratory problems. However, when COVID-19 pneumonia first strikes, those infected may initially be asymptomatic. Clinical features of pneumonia usually present after the incubation period, when patients experience mild-to-severe symptoms such as respiratory distress due to lung tissue damage. In some cases, patients do not always feel shortness of breath, even as their oxygen levels may decrease [33]. Further abnormal features such as RNAaemia, acute respiratory distress syndrome, acute cardiac injury, and sepsis can follow [31]. 

In this study, we identified several features that can be detected from a wearable NIRS sensor placed on the chest to classify respiratory patterns. These include breathing depth, breathing interval, and O_2_Hb signal amplitude. When implemented in a machine learning model, these three features together can distinguish between normal and simulated pathological breathing with a weighted average accuracy of 0.87. However, when only two of these features are used together, the accuracy of the machine learning model decreases.

Our study demonstrates the feasibility of using NIRS to monitor respiratory patterns through multiple stages of disease progression. These include screening and early diagnosis of respiratory dysfunction, continuously monitoring the respiratory rate and pattern during the infection cycle, and post-COVID rehabilitation. Although NIRS has been commercially available as a clinical device since the early 1990s, there has been limited research on the use of NIRS to monitor respiratory function. A previous study found a positive correlation between pulmonary regional oxygen saturation (rSO_2_) using NIRS and arterial partial pressure of oxygen (PaO_2_) obtained from a blood gas analysis as a gold standard [34]. A wearable NIRS sensor provides several advantages over conventional methods for monitoring respiratory function. NIRS offers continuous monitoring of respiratory rate and pattern characteristics by placing a small sensor over the chest. When combined with a machine learning model, NIRS can detect and classify different respiration patterns, providing a comfortable and rapid alternative to capnography, chest X-ray or CT scan [35]. Continuous monitoring of respiration using NIRS can be used as a regular screening method for early detection of pneumonia in high-risk individuals such as the elderly living in long-term care facilities or immune-compromised individuals. In pandemic viral outbreaks with high rates of disease transmission, at-home monitoring and diagnosis is a convenient and safe alternative, reducing exposure to the self and others [36]. Early diagnosis is essential, enabling early treatment and improved patient outcomes, and reducing long-term consequences of the disease [37]. The easy-to-use and cost-effective nature of NIRS makes a wearable sensor feasible for use in both the hospital and at home. 

For individuals admitted to the hospital with respiratory disease, monitoring gas exchange is crucial to determine the severity of the disease and response to treatment [35]. Currently, pulse oximeters are commonly used to monitor arterial oxygen saturation. However, pulse oximeters have been shown to decrease in accuracy during hypoxemic conditions, when SpO_2_ falls below 90% [9]. Furthermore, for individuals with poor peripheral perfusion or abnormal hemoglobin, pulse oximeters may display inaccurate readings [10]. Moreover, previous studies have reported that the SpO_2_ signal reported by pulse oximeters may be delayed compared to physiological changes occurring in the body [11]. NIRS sensors do not require strong pulsation to provide an accurate reading and therefore, can be used in low flow or non-pulsatile states [10]. A wearable NIRS sensor may offer a promising alternative for monitoring respiratory disease development, treatment response and determining release from the hospital.

In respiratory diseases such as COVID-19, monitoring respiratory function during the recovery period can provide invaluable information about the chronic stage of the disease and its long-term consequences. Multiple longitudinal studies have measured pulmonary function in COVID-19 patients at several time points, some up to a year post-hospitalization. Several studies using spirometry found evidence of residual pulmonary dysfunction up to six months post-hospitalization [18,19,38]. Additional studies have described abnormal radiological findings such as lung lesions after COVID-19 hospitalization [18,38,39]. Incorporating a wearable NIRS sensor into the chronic stage of the disease can contribute important information about disease severity and progression and enable better rehabilitation planning. Additionally, with continuous monitoring post-hospitalization, individuals suffering disease relapse can quickly be re-admitted to the hospital for further treatment if needed. Earlier studies have also described patient drop-out or refusal to return for re-examination following discharge from the hospital [19,39]. By monitoring respiratory function easily and non-invasively at home, NIRS alleviates many concerns that patients may have in returning to hospitals or laboratories for follow-up examinations or tests. 

A wearable NIRS sensor to monitor tissue oxygenation and respiration is just one example of the novel technologies that have been proposed for patient screening and monitoring during the COVID-19 pandemic. Several studies have proposed that using an exhaled breath condensate (EBC) device to collect samples from the respiratory tract may provide a less invasive method for screening individuals for COVID-19 [40,41,42]. By identifying specific biomarkers in the breath, EBC can potentially be used to diagnose individuals with COVID-19 [41]. Early results have been promising, with one study demonstrating the feasibility of a nanomaterial-based sensor for detecting COVID-19 from exhaled breath with strong accuracy [43]. However, due to the low viral load in breath compared to a nasopharyngeal swab, the sample collection may take up to 30 min, thus reducing the effectiveness of EBC as a rapid screening device [41]. 

Currently, there is limited research on the use of NIRS to classify respiratory patterns and monitor respiratory disease. As a result, developing a NIRS sensor for monitoring respiratory function presents a novel method for screening and monitoring respiratory disease. When combined with existing diagnostic methods such as core body temperature and SpO_2_, a multi-modal biosensor containing a NIRS sensor provides an attractive alternative to conventional diagnostic and monitoring methods. Despite our promising results, we recognize several limitations in the study, including our sample size being limited to 21 participants. Furthermore, the individuals tested in this study were healthy adults. Further studies need to be performed that include different patient cohorts such as children, neonates, immunocompromised individuals, and the elderly. Here, we used an experimental protocol to simulate symptoms commonly seen in individuals with pneumonia. Future studies can expand on our results by collecting respiratory signals from individuals with other respiratory diseases or patients admitted to the hospital with COVID-19. 

## 5. Conclusions

We demonstrated that a wearable NIRS sensor can monitor respiratory patterns continuously and non-invasively. Furthermore, we identified three respiratory features: breathing depth, breathing interval, and oxygenated hemoglobin signal amplitude, that can distinguish between normal and simulated pathological breathing as seen in people with acute pneumonia. When all three respiratory features are implemented together, a machine learning model can distinguish between normal and simulated pathological breathing with a weighted accuracy of 0.87. By monitoring respiratory function, NIRS can aid in diagnosing respiratory disease and potentially serve as a device for monitoring respiratory function and rehabilitation. 

## Figures and Tables

**Table 1:** Summary of mean accuracy using different respiratory features.

Features	Weighted Accuracy	F1 Score
Depth, interval, O_2_Hb amplitude	0.87	0.86
Depth, interval	0.79	0.77
Interval, O_2_Hb amplitude	0.78	0.76
Depth, O_2_Hb amplitude	0.63	0.62

## Data Availability

The data presented in this study are available on request from the corresponding author. The data are not publicly available due to privacy and ethical concerns.
